# Challenges and Adaptive Strategies in Transitional Care During COVID-19: A Qualitative Study of Nurses’ Experiences in Japan

**DOI:** 10.3390/nursrep15060207

**Published:** 2025-06-07

**Authors:** Yuka Sumikawa, Noriha Tanaka, Noriko Yamamoto-Mitani

**Affiliations:** 1 Department of Gerontological Home Care and Long-Term Care Nursing, Division of Health Sciences & Nursing, Graduate School of Medicine, The University of Tokyo, Bunkyo-ku, Tokyo 113-0033, Japan; noriko-tky@g.ecc.u-tokyo.ac.jp; 2 Department of Community Health Nursing, Division of Health Sciences & Nursing, Graduate School of Medicine, The University of Tokyo, Bunkyo-ku, Tokyo 113-0033, Japan

**Keywords:** COVID-19, transitional care, discharge planning, telehealth, care coordination, qualitative research

## Abstract

**Background/Objectives:** To examine shifts and challenges in transitional care practices during COVID-19 and the adaptive strategies developed by Transitional Care Nurses (TCNs) in response. **Methods**: A qualitative descriptive study was conducted using semi-structured interviews with 15 TCNs from acute care hospitals in Japan. Data were collected from October 2021 to October 2022 and analyzed using qualitative thematic analysis. The study is reported in accordance with the Consolidated Criteria for Reporting Qualitative Research (COREQ) guidelines. **Results**: Three main themes emerged: (1) disrupted connections in transitional care with patients and families, among hospital staff, and with community services; (2) professional pressures in pandemic care, including the mental and physical burden on TCNs and navigating unexpected changes to transitional care plans; and (3) adaptive strategies through digital solutions and community partnerships. TCNs implemented hybrid approaches combining selective face-to-face interactions for complex procedures and strategic use of digital tools for routine communication. **Conclusions**: This study provides the first detailed examination of how TCNs in Japan adapted to transitional care during COVID-19. TCNs maintained essential care principles while implementing digital tools and strengthening community partnerships using hybrid approaches. These findings offer practical guidance for healthcare organizations to enhance transitional care practices during future healthcare crises.

## 1. Introduction

The emergence of the coronavirus disease 2019 (COVID-19) pandemic has disrupted global healthcare systems since its emergence in December 2019. Healthcare systems faced unprecedented challenges, including resource constraints, staff shortages, and the need to rapidly adapt care processes [1,2,3]. Acute care hospitals, in particular, experienced extreme bed shortages due to the large number of patients with COVID-19. This led them to prioritize the admission of severely ill patients and to accelerate discharge processes for others [3,4,5,6,7].

Transitional care encompasses a range of interventions aimed at maintaining continuity and coordination of healthcare as patients move between settings or levels of care [8,9]. It typically includes elements such as discharge planning, coordination among healthcare providers, medication reconciliation, education for both patients and caregivers and post-discharge follow-up [9,10,11]. These processes are especially vital during transitions like hospital discharge, a period often marked by increased vulnerability to adverse events, medication discrepancies, and hospital readmissions [12,13]. Previous studies have demonstrated that well-designed transitional care programs significantly reduce 30-day readmission rates and also contribute to lowering overall healthcare costs while improving patient satisfaction and quality of life [14,15,16].

Transitional Care Nurses (TCNs) play a crucial role in bridging the gap between hospital and community care by evaluating patient needs, coordinating necessary services, delivering education, and facilitating effective communication among healthcare providers [9,17]. Their expertise is vital in recognizing patients at high risk, formulating tailored care plans, and preventing disruptions in care continuity [17,18]. The COVID-19 pandemic presented TCNs with the added challenge of swiftly adapting to evolving infection control measures, all while striving to maintain the quality of transitional care for both COVID-19 and non-COVID-19 patients [19].

While several studies have explored the experiences of acute care nurses throughout the COVID-19 pandemic [20,21], research focusing specifically on how Transitional Care Nurses (TCNs) managed the distinct challenges of providing transitional care during this period remains scarce. The pandemic undoubtedly reshaped traditional transitional care practices; however, little is known about the ways in which TCNs adapted their methods, the obstacles they faced, and the adaptive strategies they developed.

This gap in knowledge is particularly significant in the Japanese context, where the healthcare system has several unique characteristics that influenced transitional care practices during the pandemic. With one of the world’s oldest populations, Japan’s healthcare has traditionally emphasized face-to-face communication and personal relationships in care delivery, with limited use of digital technologies [22,23].

This study aims to clarify how Transitional Care Nurses in Japan navigated their practice during the COVID-19 pandemic, focusing on their lived experiences, adaptation processes, and adaptive strategies developed in response to unprecedented challenges. Through examining these experiences, we seek to uncover insights that can inform future healthcare policy development and contribute to more resilient transitional care practices during public health emergencies.

## 2. Materials and Methods

### 2.1. Research Design

We used a qualitative descriptive study using inductive thematic analysis to explore the experience of TCNs during the COVID-19 pandemic [24]. This approach was selected as it is particularly suited for examining participants’ experiences, perspectives, and behaviors in the context of previously unexplored phenomena. This study’s methodology and results are presented using the Consolidated Criteria for Reporting Qualitative Research [25].

### 2.2. Research Team and Reflexivity

The interviews were conducted by the first and second authors (YS and NT). YS is a female researcher with a PhD in Health Sciences who worked as an Assistant Professor specializing in transitional care research when this study took place. NT is a female researcher who was completing her master’s degree in transitional care research during the study. YS brought over 5 years of experience in qualitative research methods to the project, while NT contributed about 2 years of related experience. Before starting recruitment, neither researcher had formal personal or working relationships with the participants, though they recognized some initial participants as colleagues in the transitional care nursing field. We made first contact through professional networks, making sure to inform potential participants about our study goals, our backgrounds, our interest in pandemic-related transitional care, and the optional nature of participation. Throughout the study, we kept field notes where we documented our observations, key insights, and reflections on how the pandemic might have affected traditional care practices. These notes helped us track our thinking process, document important decisions, and record our developing interpretation of the interview data—all of which helped us minimize potential bias and strengthen our analysis.

### 2.3. Participants of Study

We used a combination of convenience and snowball sampling to recruit Transitional Care Nurses (TCNs) working in the Transitional Care Service Department (TCSD) of acute care hospitals in Japan. Both methods are widely used, particularly when targeting specific subpopulations or when probability sampling is not feasible [26]. We first contacted nurses through the research team’s existing professional healthcare networks and then asked these initial participants to suggest other colleagues who might be interested in participating. To be included in the study, nurses needed to (1) have at least one year of experience in transitional care, (2) have worked in TCSD during the COVID-19 pandemic, and (3) be able to communicate in Japanese. We approached 17 nurses and interviewed a total of 15 nurses for this study, as 2 nurses declined participation due to time constraints. This sample size follows recommended practices for qualitative descriptive research [27].

### 2.4. Data Collection

The target hospitals were identified through existing networks, and their participation was requested by mail to their nursing managers. TCNs who met the selection criteria were invited to participate, and written informed consent was obtained from all participants. Participants completed a brief questionnaire about their basic demographics, work experience, and role in the nursing team prior to the interview. Individual semi-structured interviews, lasting approximately 45–60 min each, were conducted from October 2021 to October 2022 based on an interview guide developed by the research team and reviewed by two external experts in transitional care. The guide was pilot-tested with two nurses who were not part of the final sample, and minor refinements were made based on their feedback. The interview guide focused on two main areas: (1) the changes/challenges in transitional care during the COVID-19 pandemic and (2) the TCN’s response. Sample questions included “How did your transitional care practice change during the COVID-19 pandemic?”, “What specific challenges did you encounter when providing transitional care?”, and “What strategies did you develop to overcome these challenges?”.

Each interview was conducted online via Zoom (Zoom Video Communications, San Jose, CA, USA) or in person in a private room at a facility designated by the participant. No repeat interviews were conducted. All interviews were audio-recorded with participants’ consent and transcribed verbatim for analysis. Field notes were taken during and immediately after each interview to capture non-verbal cues, environmental factors, and the researcher’s initial reflections.

### 2.5. Data Analysis

Following the principles of qualitative thematic analysis [24,28], we employed a systematic, iterative process. First, all recorded data were transcribed verbatim and checked for accuracy. Two researchers independently read the transcripts multiple times to gain familiarity with the data. Initial codes representing shifts, challenges, and adaptive strategies in transitional care during the pandemic were generated through open coding. These codes were then categorized by examining their similarities and differences, and broader themes were developed.

To enhance the trustworthiness of our findings, we implemented researcher triangulation by having several researchers specializing in transitional care review the coding and thematic development [29]. Additionally, we conducted member checking with three participants to validate that the findings accurately reflected their experiences. We organized and analyzed our qualitative data using Microsoft Excel for Mac (Microsoft Corporation, Redmond, WA, USA) spreadsheets, creating separate sheets for coding, categorization, and thematic development.

### 2.6. Ethical Consideration

Participants were informed verbally and in writing about the study’s purpose, methods, data handling, protection of personal information, publication of study results, and freedom to participate or withdraw from the study using a research description. Given that interviews were conducted either online using Zoom or in person due to COVID-19 restrictions, additional ethical considerations were implemented for online interviews to protect participant confidentiality and data security. For participant privacy during online interviews, we asked participants to ensure they were in private settings. Participants were informed about the digital recording process and the secure storage of interview data. For online interviews, we also confirmed that participants had adequate privacy and were comfortable with the format before beginning each session.

This study was approved by the Ethics Committee for Non-interventional and Other Research, Graduate School of Medicine, University of Tokyo (review number: 2021168NI).

## 3. Results

This study included 15 nurses. Table 1 presents the participants’ characteristics. The average work experience was 26.6 years. Of these 15 participants, 10 held administrative positions in nursing and had various leadership responsibilities in the TCSD.

Our findings revealed three main themes that directly correspond to our research aims of examining how transitional care changed during COVID-19. As Table 2 shows, we identified the following: (1) ‘disrupted connections in transitional care’, representing the shifts from pre-pandemic approaches; (2) ‘professional pressures in pandemic care’, showing the challenges TCNs faced; and (3) ‘adaptive strategies through digital solutions and community partnerships’, showing how nurses responded to these circumstances. Below, we explore each theme in detail.

### 3.1. Disrupted Connections in Transitional Care: Shifts in Pre-Pandemic Interactions

This theme captures how established care practices changed when the pandemic began. Our participants talked about breaks in connections in three main areas: with patients and their families, between hospital workers, and with community care providers.

#### 3.1.1. Disrupted Connections with Patients and Families (*n* = 14)

Almost all nurses we interviewed described problems with patient and family connections. They faced three main issues:

- Not being able to meet families face-to-face;
- Finding it hard to build trust through phone or video calls;
- Family caregivers’ inability to directly see recovery progress, leading to delayed understanding of patient’s current condition.

When hospitals restricted visitation to prevent COVID-19 spread, nurses could no longer meet with family members in person. Communication shifted to phone or video calls, making it difficult to share detailed care information effectively. Without directly seeing their family members’ recovery progress, many caregivers retained their initial impression of how ill the patient had been at admission. This gap in understanding left many families feeling unprepared when discharge was proposed. One nurse described this shift in family interactions:

*“Before visitor restrictions, families could see patients improve with their eyes. They would watch them sit up or use the bathroom with assistance. However, they could not witness any of that after the restrictions. They were left with an image of how sick the patient was when they arrived first. Therefore, when the doctor calls and says it is time to go home, the family cannot visualize what that looks like. They wonder, ‘Can I look after my loved one?’ and sometimes resists the discharge. Ultimately, we had to start by getting families on board with the idea of discharge before we could even begin preparing for it.”*

(N4)

These disruptions in family connections created substantial challenges for TCNs in their transitional care practice. Many nurses found themselves spending considerably more time convincing hesitant families about discharge readiness—families who could not witness their loved one’s recovery firsthand often had concerns about going home. This shift was so drastic that the TCN felt their routine transitional care work transformed into a totally new, formidable challenge of convincing families to discharge the client.

#### 3.1.2. Disrupted Connections Among Hospital Staff (*n* = 7)

Changes in hospital staff interactions were reported by participants. They highlighted three key shifts:

- Reduced face-to-face communication among healthcare team members;
- Adjustments to new staffing arrangements and workflows;
- Decreased efficiency in information sharing and collaboration.

In-hospital infection control measures to prevent the “three Cs” (closed spaces, crowded places, and close-contact settings) [30] significantly reduced direct interactions among hospital staff. Hospital systems and staffing arrangements frequently changed in response to infection rates, requiring staff to adapt to new workflows and communication patterns. These adjustments often resulted in less efficient information sharing and reduced collaboration. As one nurse described:

*“COVID-19 has been like an ongoing disaster for us. We had infections spreading within wards, and actually had to close some of them down. As a result, we needed to reassign nurses to different wards or outpatient departments. Even though it is the same hospital, working in an unfamiliar ward presents its challenges. It seems that even basic communication within wards became difficult.”*

(N5)

Another TCN highlighted how even informal communication or communication that happens by chance, which had previously been taken for granted in their everyday care coordination work, was affected:

*“Before COVID-19, we could have quick chats with doctors, social workers, and rehab staff during ward rounds or bumping into each other in the hallway. However, then they banned moving between wards and canceled conferences. Everyone was so busy with their work that it became difficult to coordinate, even the small staff.”*

(N6)

The breakdown in usual staff communication patterns made coordination much more difficult for TCNs. Quick hallway consultations and informal updates—previously essential for smooth transitions—were no longer possible. TCNs had to find other ways to obtain information from multidisciplinary team members and coordinate care, often through more formal channels that took significantly more time.

#### 3.1.3. Disrupted Connections with Community Services (*n* = 6)

Changes in connections with community services were reported by participants. These shifts included the following:

- Decreased direct information exchange with community care providers;
- Difficulties in arranging pre-discharge conferences;
- Challenges in building trust between families and community care staff.

The pandemic resulted in significantly decreased opportunities for direct information exchange between TCNs and community care providers or post-discharge care teams. This made it particularly difficult to communicate subtle nuances about patient care needs or to respond quickly to emergency situations. Pre-pandemic practices like pre-discharge conferences, which brought together patients, family caregivers, and community care staff to facilitate shared discharge planning, were mostly stopped. Without these face-to-face meetings, nurses found it harder to help families develop trusting relationships with the community care staff who would support them after discharge. One nurse explained:

*“The pre-discharge conferences had some great benefits for patients and their families. For instance, they could meet face-to-face with home care nurses who visit them after discharge. They also got to see us and the hospital staff, handing over information to these visiting nurses right before them. I think all of this really helped put patients and their families at ease. But now, well, it has become pretty tough to do any of that.”*

(N1)

These disruptions in community connections created substantial challenges for TCNs in ensuring continuity of care after discharge. Without face-to-face pre-discharge conferences and direct information exchange, TCNs found it difficult to facilitate trust-building between families and community care providers. TCNs thus faced the additional challenge of establishing these crucial relationships through remote methods.

### 3.2. Professional Pressures in Pandemic Care: Challenges Faced by TCNs

This theme represents the significant challenges TCNs encountered during the pandemic. Participants described two major areas of challenge: the mental and physical burden they experienced, and the complexities of managing unexpected changes to transitional care plans.

#### 3.2.1. Mental and Physical Burden on TCNs (*n* = 9) 

Changes in how care was delivered created substantial strain for these nurses. The key challenges included:

- Inability to provide the same level of detailed care as before the pandemic;
- Professional distress when pandemic restrictions prevented delivering care to their usual standards;
- Increased workload due to staffing shortages and new infection control procedures.

TCNs typically maintain close relationships with patients and their families and conduct physical and mental care through direct contact and dialogue. Under visiting restrictions, many nurses found it impossible to provide the same level of detailed care as before, leading to significant mental strain. They experienced conflict and a sense of inadequacy when unable to deliver care according to their professional standards. Additionally, increased workload due to staffing shortages and new infection control procedures contributed to physical exhaustion, creating a compounded burden during the pandemic. One TCN reflected on this challenge:

*“I think transitional services should prioritize detailed communication to establish trust and work with families and patients so that they can safely move on in wherever their next care setting is. However, with the time crunch we were under, our conversations with patients and families started to feel like we were just trying to extract the information we needed for discharge.”*

(N4)

TCNs had to undertake a higher volume of work as hospitals faced a larger workforce shortage due to staff infections, quarantine protocols, and other pandemic-related staffing challenges. The increased stress caused by this pandemic created a longer and higher level of mental strain and physical exhaustion on TCNs.

#### 3.2.2. Navigating Unexpected Changes to Transitional Care Plans (*n* = 11)

Many TCNs shared experiences of having to constantly revise their care plans. They faced several difficulties:

- Community services shutting down or changing their rules, forcing plan changes;
- Pressure from both hospitals needing beds and community places refusing patients;
- Trouble finding safe places for patients to go during COVID-19 outbreaks.

When the pandemic hit, many community services either closed or changed how they operated. Community care providers started refusing to take new patients after discharge, forcing nurses to frequently revise the plans they had made. Despite these adjustments, getting patients discharged on time often remained a struggle.

Hospitals needed to make room for COVID-19 patients while also trying to safely discharge others. This put nurses in a difficult position, as one explained:

*“Even though there were not many places to transfer patients to, we often encountered problems. We would finally find a place willing to take a patient, but then a COVID case would pop up in the same ward. Even if our patients tested negative, the new facility often refused to take them. This was happening all the time.”*

(N11)

Another TCN described feeling caught in the middle:

*“Our hospital is an emergency hospital; therefore, our top priority was to make room for more critical patients. Because of this, we were under a lot of pressure to discharge less severely ill patients. However, it is not that simple. Local facilities were often at full and had to turn patients away. We were caught between a rock and a hard place.”*

(N8)

### 3.3. Adaptive Strategies Through Digital Solutions and Community Partnerships: TCNs’ Practical Approach

This theme shows how TCNs responded to the limitations on face-to-face care and community resource disruptions during the pandemic. The TCNs in this study came up with various approaches to keep delivering quality care: holding onto essential care practices, using new digital tools, and finding ways to work with community partners.

#### 3.3.1. Maintaining Essential Pre-Pandemic Transitional Care Elements (*n* = 11)

Even with pandemic restrictions, many TCNs worked to preserve essential components of transitional care. They adapted several approaches:

- Prioritizing which care components were critical and could not be compromised, even when difficult decisions had to be made;
- Adapting their information-gathering methods to be more efficient;
- Offering more organized guidance to help families navigate the discharge process,

When faced with new limitations, TCNs needed to identify priorities in care delivery: what needed to be preserved and what could be modified. They became more strategic about collecting patient information and created clearer pathways for the entire discharge journey. Throughout these changes, they maintained their focus on helping families understand their loved one’s condition and preparing them for discharge, even when the process had to look different.

For certain essential care activities that could not be carried out remotely—such as demonstrating medical procedures or providing hands-on guidance—TCNs would arrange face-to-face meetings with careful safety protocols. One TCN described this selective approach:

*“I carefully selected patients who needed face-to-face discussions and conducted in-person conferences and skill training with thorough infection control measures in place.”*

(N15)

#### 3.3.2. Adopting and Implementing Digital Solutions in Transitional Care (*n* = 13)

Faced with visitor limitations and infection control requirements, many TCNs incorporated digital technologies into their practice. They utilized digital tools in several ways:

- Moving care conferences online to share information across distances;
- Using visual materials like photos and videos to ensure care consistency;
- Adapting common communication technologies to connect patients with their families remotely.

TCNs evaluated various digital tools and determined where they could be most effectively applied in transitional care. The most notable adoption was the shift to online conferences, which enabled efficient information sharing across geographical distances. These remote collaboration platforms facilitated the coordination of patient care despite physical separation. TCNs also implemented these digital tools to maintain secure channels for patient-to-family communication when in-person visits were not possible.

Many TCNs found unexpected benefits in these digital adaptations, as one TCN explained:

*“We had not been conducting online family consultations, but when we started doing them out of necessity, we found some unexpected benefits. Even without visitation restrictions, it is very convenient to share information in real-time with family members who live far away.”*

(N12)

TCNs also enhanced their use of visual information. They ensured care consistency by providing information to family caregivers and community service providers using photos and videos of care procedures. One TCN described this approach:

*“We recorded patients performing stoma care while explaining how to care. This helps the family caregiver to assist at home by viewing the video again. We have found that keeping visual records of medical instructions is a great way to support patients and their families.”*

(N3)

While these digital solutions offered many advantages, they also presented challenges. Some older adults and those with limited technology experience found device operation difficult, making online visits and counseling less effective in certain cases:

*“When it comes to online consultations, using the devices can be a real challenge. Even if we healthcare workers can handle Meet or Zoom, it is a different story whether the families can use them the same way.”*

(N10)

#### 3.3.3. Promoting Community Service Reopening (*n* = 6)

In response to the severe shortage of community resources, some TCNs extended their scope of practice to actively rebuild support networks beyond the hospital. Their approaches included the following:

- Collaborating with local public health authorities to safely restore services;
- Providing infection control education to community care providers;
- Helping establish new protocols for safe patient transfers between facilities.

TCNs worked with local public health officials and neighboring medical institutions to develop strategies that would allow community services to safely resume operations. This collaborative effort strengthened the framework within the community and created pathways for smoother patient transfers and service reinstatement.

One TCN described establishing a specialized team to address transfer challenges:

*“To take in new patients with COVID-19, we needed to transfer those past the acute phase, but the process was still not going smoothly. Therefore, we set up a ’Patient Transfer Coordination Team’ involving the local public health center, nearby hospitals, and even temporarily closed facilities to streamline transfers.”*

(N8)

TCNs provided infection prevention guidance to community service providers who had either suspended operations due to COVID-19 concerns or were struggling to maintain services safely during the pandemic. They shared current information on protective measures and kept providers updated on frequently changing infection control guidelines:

*“Since many of these local service providers are not medical professionals, I tried to provide information that is easy for the general public understand. I actively shared the latest information on things like when and how to wash hands and wear masks and what to watch out for if you are in close contact.”*

(N12)

Another TCN described a direct approach to information sharing:

*“We set up opportunities for our ward nurse staff to directly hand over infection control information to facility staff and local service providers.”*

(N14)

## 4. Discussion

The COVID-19 pandemic fundamentally challenged the relational foundation of transitional care, transforming how healthcare professionals navigate the complex coordination between hospital discharge, family preparation, and community integration. Our findings show that TCNs experienced significant changes in their professional roles, expanding from their traditional patient-centered focus to include infection control education, community coordination, and digital technology implementation. This transformation reflects broader changes in nursing roles during health emergencies, where professionals must rapidly adapt their practice while maintaining care quality under unprecedented constraints.

The framework of shifts, challenges, and adaptive strategies that emerged from our analysis helps us understand how healthcare professionals maintain care continuity during system-wide disruptions. Unlike previous studies that focused primarily on nursing within acute care hospital units during COVID-19 [31,32], our research shows the unique position of TCNs who must coordinate across multiple systems and stakeholders while managing the critical transition from hospital to community care. This coordination role became particularly complex when face-to-face interactions were restricted, requiring TCNs to maintain essential family trust and community partnerships through remote methods. The adaptive strategies identified in our study suggest that successful adaptation in transitional care during disruptions requires both individual professional flexibility and the ability to effectively use technology and strengthen community partnerships under pressure. These findings contribute to recent studies examining healthcare system adaptation and professional responses during pandemic conditions [33,34].

### 4.1. Disrupted Connections in Transitional Care: Multifaceted Challenges and Implications

The disrupted connections identified in our study reveal fundamental challenges to the relational foundation of transitional care, where trust-building and direct communication are central to effective practice. When established communication pathways were disrupted, TCNs experienced a forced redefinition of their professional relationships with patients, families, and community partners. Previous studies have pointed out the decline in family understanding and acceptance of the patient’s situation and care due to visitation restrictions [35]. In the present study, it became clear that the reason for the lack of progress in family understanding even when information was provided via telephone and other ICT methods is that, with non-face-to-face communication such as telephone calls, only fragmented information is conveyed every few days, making it difficult for the family to make decisions based on the information obtained daily. Under visitation restrictions, the ability of TCNs to explain the patient’s progress, which is based on an assessment of the family’s ability to understand and convey the patient’s progress in words, is thought to significantly impact the family’s level of understanding.

Our study also highlights how disrupted connections among hospital staff impacted care delivery. Enhanced infection control measures during COVID-19 significantly disrupted communication and collaboration between multidisciplinary teams in hospitals, which supports the findings of Westbrook et al. [36] and Rehder et al. [37], who found changes in teamwork and cooperation among hospital staff during the pandemic, and Jordan et al. [38], who identified challenges in interprofessional communication and care coordination among multidisciplinary teams during COVID-19.

### 4.2. Professional Pressures in Pandemic Care: Mental Burden and Navigating Care Plan Changes

The mental and physical burden on TCNs was a significant aspect of professional pressures during the pandemic. Navigating the difficult balance between providing high-quality transitional care and strict infection prevention protocols created substantial challenges. TCNs felt conflicted and inadequate when they were unable to meet the needs of patients and families, such as being unable to provide in-person emotional support, not facilitating face-to-face consultation with family members, or not directly providing detailed discharge instructions. This conflict between professional expectations and practice constraints has been identified as a significant source of distress among healthcare workers in coordination roles [39]. The challenge of managing unexpected changes to transitional care plans created dual pressures, with TCNs caught between hospital demands for rapid discharge and community services that were unavailable or reluctant to accept patients. This reflects broader healthcare system strain during COVID-19, where workers faced unprecedented demands with reduced resources [40].

Our findings also reveal how TCNs’ roles expanded beyond traditional patient-centered care to include infection control education and community service coordination. While this adaptability demonstrates professional resilience, research suggests that role expansion without adequate support can contribute to burnout [41]. These expanded roles require organizational recognition and support rather than being treated as temporary pandemic adjustments.

### 4.3. Adaptive Strategies Through Digital Solutions and Community Partnerships: Innovations for Future Practice

The adaptive strategies developed by TCNs during the pandemic represent more than temporary adjustments—they reflect fundamental shifts in how transitional care can be delivered when traditional approaches are disrupted. Our findings reveal that TCNs demonstrated remarkable professional adaptability by maintaining core care principles while integrating new technologies and expanding community coordination roles. The adoption of digital solutions in transitional care emerged as a critical adaptive strategy, with TCNs utilizing online conferences, visual documentation, and remote family consultations to bridge communication gaps created by visitor restrictions. These digital adaptations align with broader trends in healthcare technology adoption accelerated by the pandemic [42]. However, our participants also identified important limitations, particularly challenges with technology literacy among older patients and families, highlighting the need for inclusive digital health approaches that consider diverse user capabilities.

These adaptive strategies provide valuable insights for building more resilient transitional care systems. The selective use of face-to-face interactions for complex procedures and trust-building, combined with strategic digital tool implementation, offers a framework for hybrid care models that could enhance transitional care delivery beyond the pandemic context.

This study has several limitations. First, we employed convenience and snowball sampling methods, which may have introduced selection bias and resulted in participants with similar backgrounds or perspectives. We interviewed participants at a single time point rather than tracking how their experiences changed throughout the pandemic. A significant limitation relates to our data collection method, as 80% of interviews (*n* = 12) were conducted online rather than in person. While this approach enabled data collection during pandemic restrictions, it may have limited our ability to observe participants’ reactions and emotions directly, which could have enriched our understanding of their experiences. Online interviews may have also affected the rapport between researchers and participants, potentially influencing the depth of shared experiences.

Our study focused exclusively on TCNs, and we could not interview patients, families, community service providers, and other healthcare professionals. This single perspective limits our ability to present a comprehensive view of how pandemic disruptions affected the entire transitional care process. Additionally, the Japanese healthcare context may limit the generalizability of findings to other countries with different healthcare structures or digital technology adoption levels. Finally, while we employed member checking with three participants to validate our themes, we did not return full transcripts to all participants for review. When participants described their pre-pandemic practices, these accounts may have been influenced by recall bias, as their memories could have been affected by their recent pandemic experiences.

Despite these limitations, this focused approach allowed us to gain deep insights from frontline professionals who experienced transitional care before and during the pandemic. Understanding TCNs’ unique perspectives is critical, as they occupy a key position connecting hospital care, patient and family needs, and community services [9]. Their comprehensive view provides valuable foundational knowledge for future research including other stakeholders.

## 5. Conclusions

This study provides the first detailed examination of how TCNs in Japan adapted their practice during the COVID-19 pandemic. Using a framework of shifts, challenges, and adaptive strategies, we documented how TCNs’ roles expanded significantly beyond traditional patient-centered care during the pandemic. A key contribution is the identification of specific situations requiring face-to-face interactions during crisis conditions, including complex medical procedures and trust-building with new care providers. This specificity addresses gaps in pandemic care guidelines that typically offer only general visitor restrictions. For practice, our findings suggest healthcare organizations should develop hybrid communication strategies combining digital tools with selective face-to-face interactions based on clinical necessity. These findings provide valuable insights for improving transitional care resilience during future healthcare crises while maintaining quality patient outcomes.

Future research should include perspectives from patients, families, and community providers, examine long-term effects of pandemic adaptations, and explore optimal integration of digital technologies in transitional care. Comparative studies across different healthcare systems would enhance understanding of universally applicable versus culturally specific strategies.

## Figures and Tables

**Table 1:** Characteristics of participants.

No	Age	Gender	Work Experience, Years	Role of Nursing Team	Education
N1	40–49	Female	20	Staff nurse	Associate
N2	50–59	Female	33	Nurse leader	Diploma
N3	50–59	Female	35	Nurse leader	Master
N4	60–69	Female	40	Nurse leader	Master
N5	50–59	Female	33	Nurse leader	Diploma
N6	40–49	Female	19	Nurse leader	Diploma
N7	50–59	Female	24	Nurse leader	Diploma
N8	50–59	Female	30	Nurse leader	Master
N9	30–39	Female	15	Staff nurse	Diploma
N10	50–59	Female	33	Nurse leader	Master
N11	40–49	Female	18	Nurse leader	Master
N12	50–59	Female	26	Staff nurse	Master
N13	40–49	Female	20	Staff nurse	Master
N14	50–59	Female	35	Nurse leader	Diploma
N15	40–49	Female	19	Staff nurse	Diploma

**Table 2:** Research framework elements, themes, and categories.

Framework Element	Themes	Categories (Number of Participants, N = 15)
Shifts	Disrupted connections in transitional care	Disrupted connections with patients and families (n = 14)
		Disrupted connections among hospital staff (n = 7)
		Disrupted connections with community services (n = 6)
Challenges	Professional pressures in pandemic care	Mental and physical burden on TCNs (n = 9)
		Navigating unexpected changes to transitional care plans (n = 11)
Adaptive Strategies	Adaptive strategies through digital solutions and community partnerships	Maintaining essential pre-pandemic transitional care elements (n = 11)
		Adopting and implementing digital solutions in transitional care (n = 13)
		Promoting community service reopening (n = 6)

Abbreviations: TCN, transitional care nurse.

## Data Availability

The de-identified data underlying the results presented in this study may be available from the corresponding author upon reasonable request, subject to privacy considerations and institutional approval processes. Any shared data will be in a form that does not compromise participants’ confidentiality or privacy.

## Public Involvement Statement

There was no public involvement in any aspect of this study.

## Guidelines and Standards Statement

This manuscript was drafted according to the Consolidated Criteria for Reporting Qualitative Research.

## Use of Artificial Intelligence

DeepL (DeepL SE, Colonia, Germany) and Claude (Anthropic, San Francisco, CA, USA) were used for initial translation of interview data from Japanese to English. To ensure translation accuracy and conceptual equivalence, all AI-assisted translations underwent a back-translation process and were reviewed by bilingual researchers. Grammarly (Grammarly, Inc., San Francisco, CA, USA) was used to check grammar and spelling, with all suggestions manually reviewed by the authors for accuracy in the academic healthcare context.

## Abbreviations

The following abbreviations are used in this manuscript:

COVID-19	Corona virus disease 2019
ICT	Information and communication technology
TCN	Translational Care Nurse
TCSD	Transitional Care Service Department
